# The Role of PGE_2_ in Alveolar Epithelial and Lung Microvascular Endothelial Crosstalk

**DOI:** 10.1038/s41598-017-08228-y

**Published:** 2017-08-11

**Authors:** Thomas Bärnthaler, Jovana Maric, Wolfgang Platzer, Viktoria Konya, Anna Theiler, Carina Hasenöhrl, Benjamin Gottschalk, Sandra Trautmann, Yannick Schreiber, Wolfgang F. Graier, Rudolf Schicho, Gunther Marsche, Andrea Olschewski, Dominique Thomas, Rufina Schuligoi, Akos Heinemann

**Affiliations:** 1 Institute of Experimental and Clinical Pharmacology, Medical University of Graz, Graz, Austria; 2 Institute of Molecular Biology and Biochemistry, Medical University of Graz, Graz, Austria; 3 Ludwig Boltzmann Institute Lung Vascular Research, Graz, Austria; 4 Pharmazentrum Frankfurt/ZAFES Institute of Clinical Pharmacology, Goethe-University Frankfurt, Frankfurt, Germany; 5 Fraunhofer Institute for Molecular Biology and Applied Ecology IME, Project Group TMP, Frankfurt, Germany

## Abstract

Disruption of the blood-air barrier, which is formed by lung microvascular endothelial and alveolar epithelial cells, is a hallmark of acute lung injury. It was shown that alveolar epithelial cells release an unidentified soluble factor that enhances the barrier function of lung microvascular endothelial cells. In this study we reveal that primarily prostaglandin (PG) E_2_ accounts for this endothelial barrier-promoting activity. Conditioned media from alveolar epithelial cells (primary ATI-like cells) collected from BALB/c mice and A549 cells increased the electrical resistance of pulmonary human microvascular endothelial cells, respectively. This effect was reversed by pretreating alveolar epithelial cells with a cyclooxygenase-2 inhibitor or by blockade of EP4 receptors on endothelial cells, and in A549 cells also by blocking the sphingosine-1-phosphate_1_ receptor. Cyclooxygenase-2 was constitutively expressed in A549 cells and in primary ATI-like cells, and was upregulated by lipopolysaccharide treatment. This was accompanied by enhanced PGE_2_ secretion into conditioned media. Therefore, we conclude that epithelium-derived PGE_2_ is a key regulator of endothelial barrier integrity via EP4 receptors under physiologic and inflammatory conditions. Given that pharmacologic treatment options are still unavailable for diseases with compromised air-blood barrier, like acute lung injury, our data thus support the therapeutic potential of selective EP4 receptor agonists.

## Introduction

Pulmonary gas exchange in the lung is accomplished by the alveolar-capillary structure, which consists of an endothelial and an alveolar epithelial cell barrier with their individual basement membranes fused together in order to facilitate diffusion^1^. There are two types of alveolar epithelial cells: type I (ATI) cells which cover around 95% of alveolar surface and are considered to be crucial in the barrier function, and cuboidal type II (ATII) cells, which cover 3–5% of the alveolar surface and play a role in immune responses and release surfactants^2–4^. Under certain conditions, ATII cells can undergo transdifferentiation towards ATI cells, thus protecting barrier integrity^5, 6^. This is mimicked by the changes undergone by isolated ATII cells, which - if kept in culture - become ATI-like cells^7^.

Endothelial cells lining the pulmonary microvessels are in proximity to the alveolar epithelial cells. They form a tight barrier, characterized by adherens (e.g. VE-cadherin) or tight junction (e.g. occludin) proteins and high resistance to ion flux^8^. Furthermore, they mediate immune cell trafficking and play a key role in immunomodulation^9^.

Acute lung injury (ALI), or acute respiratory distress syndrome (ARDS) are syndromes defined by inflammation, compromised lung function and increased vascular permeability. It is a response of the lung to infectious, inflammatory or chemical insults as well as trauma^10^. The disruption of the blood-air barrier followed by inflammatory cell influx and edema formation is an important hallmark of ALI/ARDS^9^. Despite major efforts, mortality rate of ARDS patients is still around 30–50%, as adequate pharmacologic treatment is not yet available^10, 11^. Thus, targeting endothelial barrier maintenance is in the focus of ongoing research. Various *in vivo* models are applied to gain a broader understanding of relevant aspects^12^. The aim of this study is to unravel key mechanisms, which could be exploited as approaches for novel treatment options.

As endothelial and epithelial cells in the lung are located in proximity, there have been studies concerning their crosstalk and interaction, especially in terms of barrier function regulation. It has been shown that endothelial cells contribute to the protection of alveolar epithelial integrity^13^. However, other studies report about endothelial barrier disrupting factors derived from alveolar epithelial cells^14^. A recent study revealed that conditioned medium from A549, as well as from human alveolar epithelial cells, enhances endothelial barrier function and that the effect was maintained in the lipid fraction^15^. Nevertheless, the responsible agent(s) could not be unraveled.

A549 cells produce PGE_2_^16, 17^, which plays important roles in the lung^18^ and, as we have shown recently, PGE_2_ promotes lung microvascular integrity^19^. Therefore, we hypothesized that PGE_2_ could account for that epithelium-derived, barrier-promoting mediator in question. PGE_2_ is an arachidonic acid metabolite and exerts its effects by binding to four different G protein coupled receptors (EP1-EP4) which have tissue-specific distribution. We found previously that PGE_2_ enhances barrier function in pulmonary human microvascular endothelial cells (HMVEC-L) via EP4 receptor activation^19^ and exerts beneficial effects in a mouse model of LPS-induced ALI^20^. There are indications that isolated alveolar epithelial cells from mice express cyclooxygenase (COX)−1 and COX-2, synthesize PGE_2_ via COX-2 pathway^21^ and that COX-2 is present in human type II alveolar epithelial cells^22^. Therefore, the aim of this study was to investigate whether PGE_2_ – synthesized by the alveolar epithelium - plays a role in regulating the microvascular barrier. Indeed, by using the human epithelial cell line A549 as well as primary mouse alveolar type (AT) I-like cells we found that primary alveolar epithelial cells constitutively express both COX isoforms, while A549 express only COX-2. Additionally, we revealed that PGE_2_, via activating endothelial EP4 receptors, is the major endothelial barrier-regulating mediator released by alveolar epithelial cells under both physiologic and inflammatory conditions.

## Results

### Alveolar epithelial cells release PGE_2_

**Figure 1:**
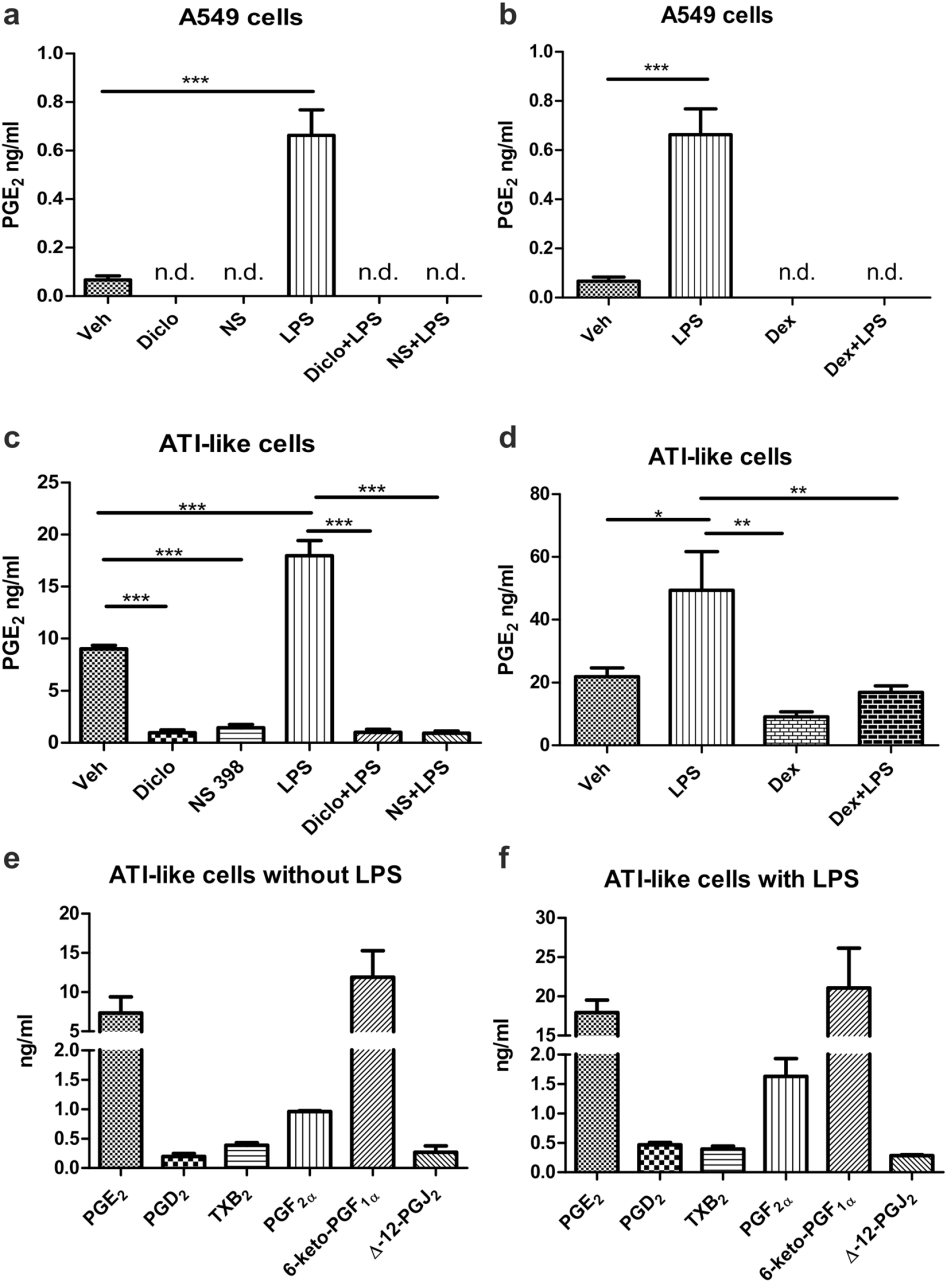
A549 cells as well as primary mouse ATI-like cells release PGE_2_. (**a**,**b**) A549 cells were kept in culture for 3 days. On the third day, cells were pretreated (**a**) with either diclofenac (diclo, 10 µM), NS398 (1 µM), or vehicle (Veh) for 20 min; (**b**) dexamethasone-pretreatment (dexa 1 µM) was performed for two days. Thereafter, cells were stimulated with LPS (10 µg/ml) or vehicle (Veh). (**c,d,e,f**) Primary ATI-like cells were isolated from BALB/c mice and kept in culture for 6 days. After 6 days, medium was changed and cells were pretreated with (**c**) either diclofenac (diclo, 10 µM), NS398 (1 µM), or vehicle (Veh) for 20 min; (**d**) dexamethasone-pretreatment (dexa 1 µM) was performed for three days. Thereafter, cells were stimulated with LPS (10 µg/ml) or vehicle (Veh). n.d. not detectable. (**e,f)** Primary mouse ATI-like cells in addition to PGE_2_ secrete a broad spectrum of prostaglandins under both resting and LPS-stimulated conditions as determined by mass spectrometry (n = 3). Statistical analysis was performed using **(a,b**) two-tailed t-test for A549 (n = 6) or (**c,d**) One Way ANOVA and Bonferroni’s post-hoc test for ATI-like cells (n = 5), *p < 0.05; **p < 0.01; ***p < 0.001.

First we investigated whether A549 and mouse ATI-like cells release PGE_2_. We found low amounts of PGE_2_ (0.067 ± 0.017 ng/ml; Fig. 1a) in the supernatants of A549 cells after 24 hours but PGE_2_ release increased when cells were stimulated with LPS (10 µg/ml) for 24 hours (Fig. 1a,b). Pretreatment with dexamethasone (1 µM), or the selective COX-2 inhibitor NS398 (1 µM) and the non-selective COX inhibitor diclofenac (10 µM) totally prevented PGE_2_ release, both in the presence and absence of LPS (Fig. 1a,b). Interestingly, dexamethasone abolished the release of PGE_2_ in these cells already at baseline. Primary mouse ATI-like cells showed release of high amounts of PGE_2_ under basal conditions (9.0 ± 0.3 ng/ml; Fig. 1c). Release was increased approximately two-fold upon LPS-stimulation (10 µg/ml) for 8 hours (Fig. 1c,d). Diclofenac (10 µM) and NS398 (1 µM) considerably diminished PGE_2_ release, both in the absence and presence of LPS (Fig. 1c). In addition, we observed that dexamethasone inhibited the LPS-induced increase of PGE_2_, but failed to reduce baseline release in primary ATI-like cells (Fig. 1d). Furthermore, we found that primary AT-I like cells, but not A549 cells in addition to PGE_2_ release high amounts of the PGI_2_ metabolite 6-keto-PGF_1α_ (11.91 ± 3.37 ng/ml), while low levels of PGF_2α_ (0.96 ± 0.01 ng/ml), PGD_2_ (0.20 ± 0.05 ng/ml) and the PGD_2_ metabolite Δ-12-PGJ_2_ (0.27 ± 0.13 ng/ml) and trace amounts of the stable thromboxane metabolite, TXB_2_ (0.38 ± 0.04 ng/ml) were detected by mass-spectrometry under resting conditions. LPS stimulation (10 μg/ml) caused an increase in prostanoid concentrations, which, besides PGE_2_, reached significance for PGD_2_ and 6-keto-PGF_1α_ (p = 0,029 and p = 0.042 respectively; n = 3) (Fig. 1e,f).

### Alveolar epithelial cells express COX-2 which is increased upon LPS stimulation

**Figure 2:**
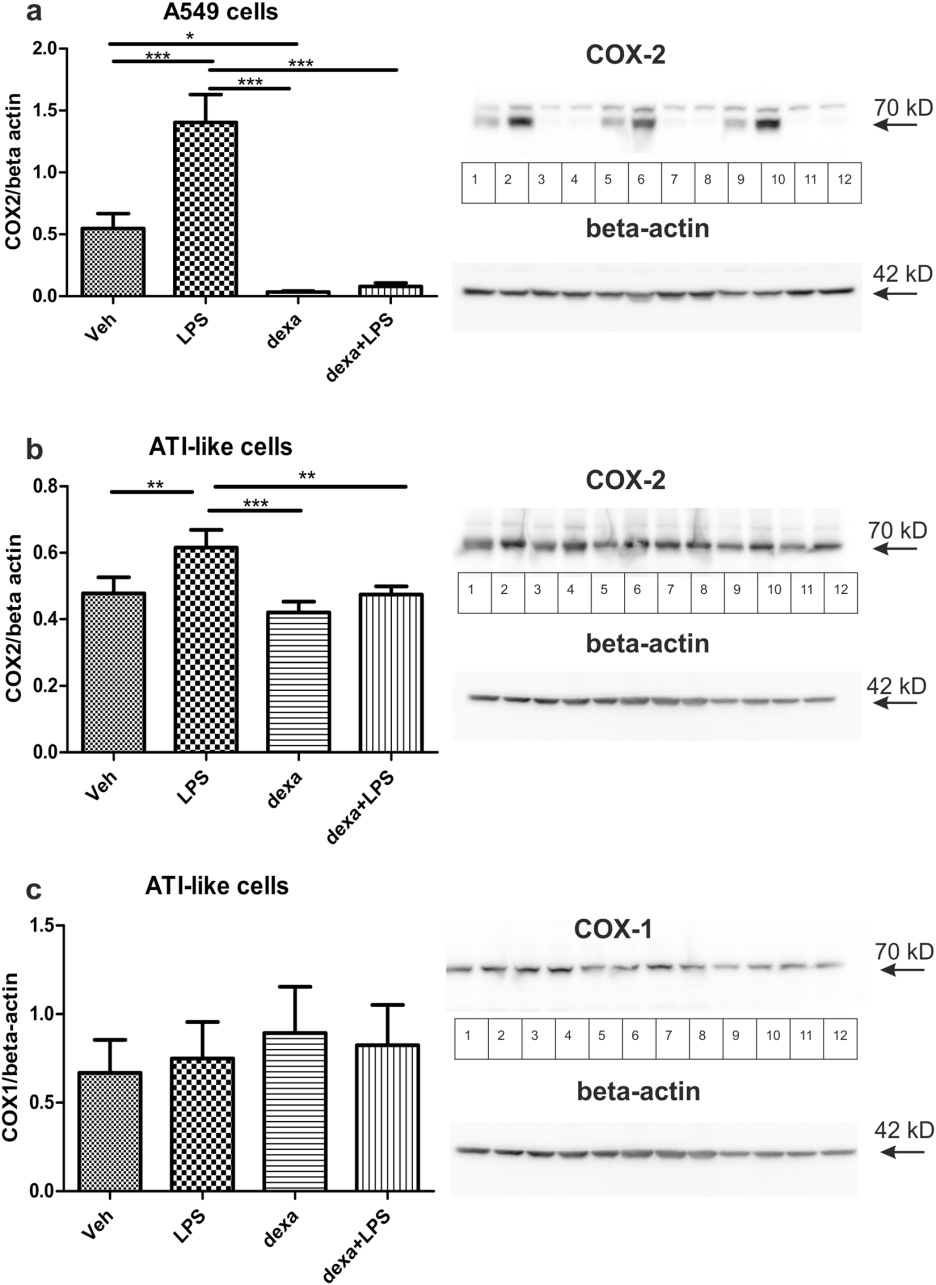
Primary mouse ATI-like cells express COX-2 and COX-1 and A549 cells only COX-2. (**a**) A549 or (**b**,**c**) mouse primary ATI-like cells were treated with vehicle (Veh), LPS (10 µg/ml) and/or dexamethasone (dexa; 1 µM) and (**a,b**) COX-2 or (**c**) COX-1 expression was determined by Western blot. A549 cells did not express COX-1. Densitometric values normalized to beta actin are shown as mean + SEM. Inserts on the right show sections of typical blots (lanes vehicle: 1,5,9; LPS: 2,6,10; dexamethasone: 3,7,11; LPS + dexamethasone: 4,8,12). The inserts in (**b**) and (**c**) show the same membrane and thus the same loading control was used. n = 6 for A549 cells and n = 5 for primary ATI-like cells. Statistical analysis was performed using One Way ANOVA and Bonferroni’s post-hoc test, *p < 0.05; **p < 0.01; ***p < 0.001.

Since we found that the COX-2 inhibitor NS398 reduced PGE_2_ release already under basal conditions, *i.e*. in the absence of LPS, we investigated whether these cells express COX-2 protein. In A549 cells, COX-2 was present in vehicle-treated cells and its expression was approximately three-fold increased upon 24 hours of LPS-stimulation (10 µg/ml). Interestingly, dexamethasone (1 µM) inhibited baseline as well as LPS-induced expression of COX-2 (Fig. 2a), which is in line with our findings showing that dexamethasone also inhibited basal PGE_2_ release. We were unable to detect COX-1 in A549 cells. Since A549 is an adenocarcinoma cell line, we were interested whether isolated primary mouse ATI-like cells constitutively express COX-2. In fact, we found that these cells also express COX-2 already under unstimulated conditions (Fig. 2b). LPS-stimulation (10 µg/ml) for 8 hours increased COX-2 expression in ATI-like cells 1.3 fold, which was blocked by pretreatment with dexamethasone (1 µM). However, dexamethasone only inhibited the LPS-induced increase of COX-2 expression, suggesting constitutive expression of COX-2 (Fig. 2b). COX-1 is also expressed in ATI-like cells, but was not altered upon treatment (Fig. 2c).

### Mouse ATII cells constitutively express COX-1 and COX-2

**Figure 3:**
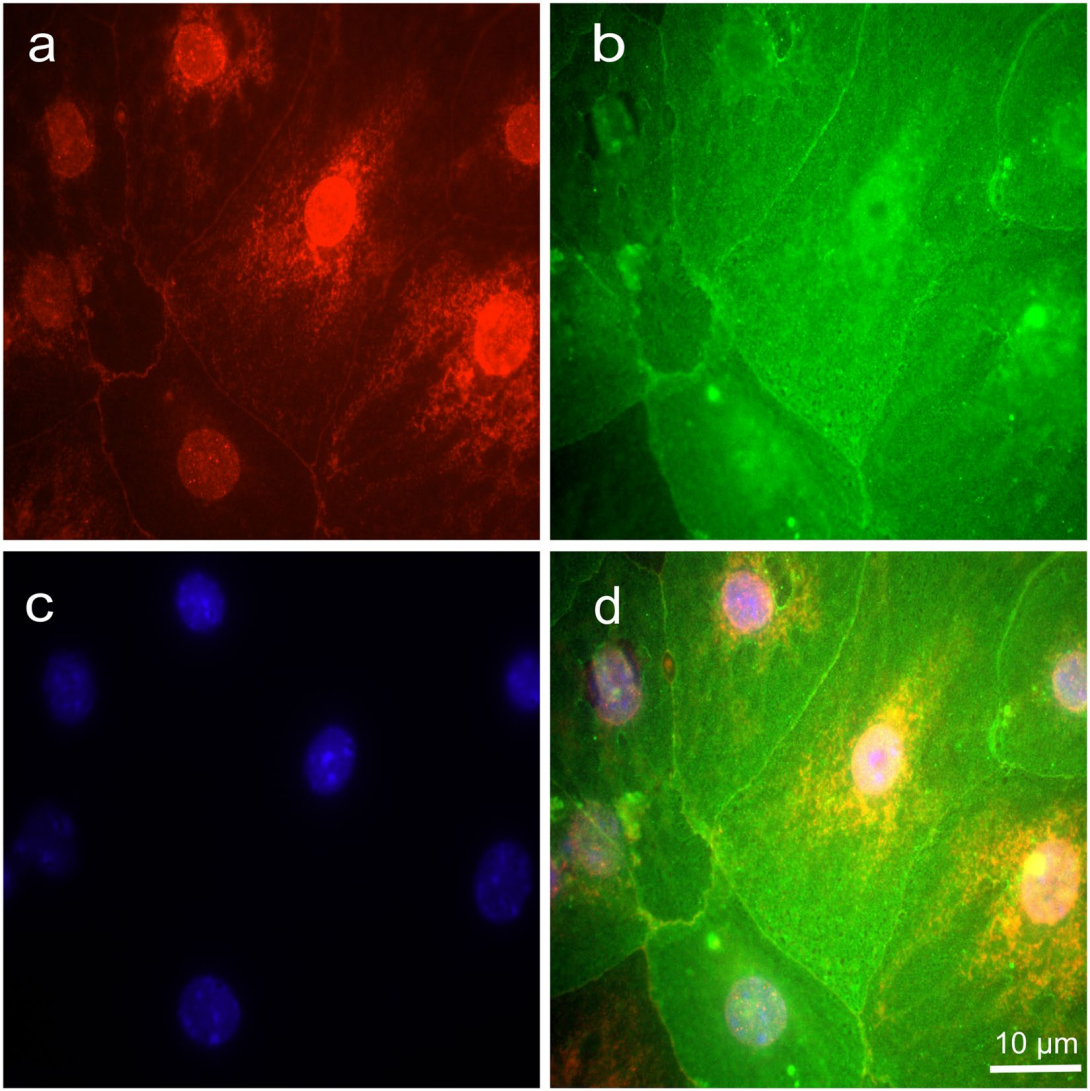
Primary mouse ATI-like cells express COX-2. Cells grown in chamber slides for 6 days were stained with antibodies directed (**a**) against COX-2 (red), followed by incubation with (**b**) an anti-AQP5 antibody (green), a marker of ATI-cells. (**c**) Samples were counterstained with DAPI (blue) to reveal localization of nuclei; (**d**) shows the overlay. Images are representative for 5 independent experiments.

In addition, we observed by immunofluorescence that mouse ATI-like cells (cultured for six days), express COX-1 and, most markedly, also COX-2. COX-1 was primarily visible in the perinuclear region (see Supplementary Fig. S1). However, COX-2 immunofluorescence seems to be distributed distinctly throughout the cytoplasm (Fig. 3). 97.6% ± 1.5% of cultured cells stained positive for AQP5 (see Supplementary Fig. S2), a marker of ATI -like cells and again 97.5 ± 2.5% of those cells stained positive for COX-2 (n = 5).

**Figure 4:**
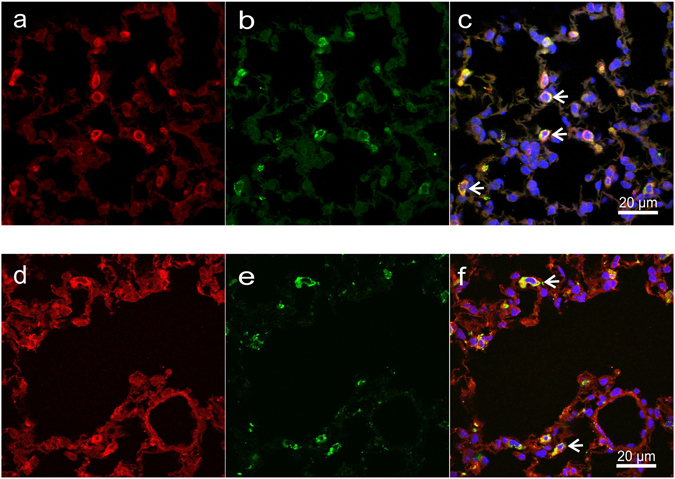
Lung ATII cells express COX-2 and COX-1. Lung sections from healthy, specific pathogen-free BALB/c mice were stained with an antibody detecting (**a**) COX-2 or (**d**) COX-1 (red) followed by immunodetection of (**b,e**) pro-surfactant protein C, a marker of type II alveolar epithelial cells (green). The samples were counterstained (**c,f**) with DAPI in order to visualize the nuclei. The overlays show that (**c**) COX-2 as well as (**f**) COX-1 is expressed in ATII cells (e.g. arrows). Images are representative for 4 independent experiments.

To exclude the possibility that COX-2 expression might have been induced due to the isolation procedure of the primary cells, we investigated the expression of COX-1 and COX-2 in sections of the lung from specific pathogen free BALB/c mice by immunofluorescence. We found that COX-2 is present in ATII cells, identified by pro-surfactant protein C (SPC), even in mice without any ongoing inflammation (Fig. 4), which confirms our results of constitutive expression of COX-2 in primary mouse ATI-like cells. COX-1 was also present in ATII cells to some extent; however, its cellular distribution pattern was more even throughout the lung when compared with the COX-2 staining (Fig. 4). This was represented by the fact that pro-SP-C positive cells accounted for 84 ± 7.6% of all COX-2 positive cells but only for 18.2 ± 2.8% of COX-1 positive cells.

### Conditioned medium from A549 cells induces endothelial barrier enhancement via EP4 and S1P_1_ receptor activation

**Figure 5:**
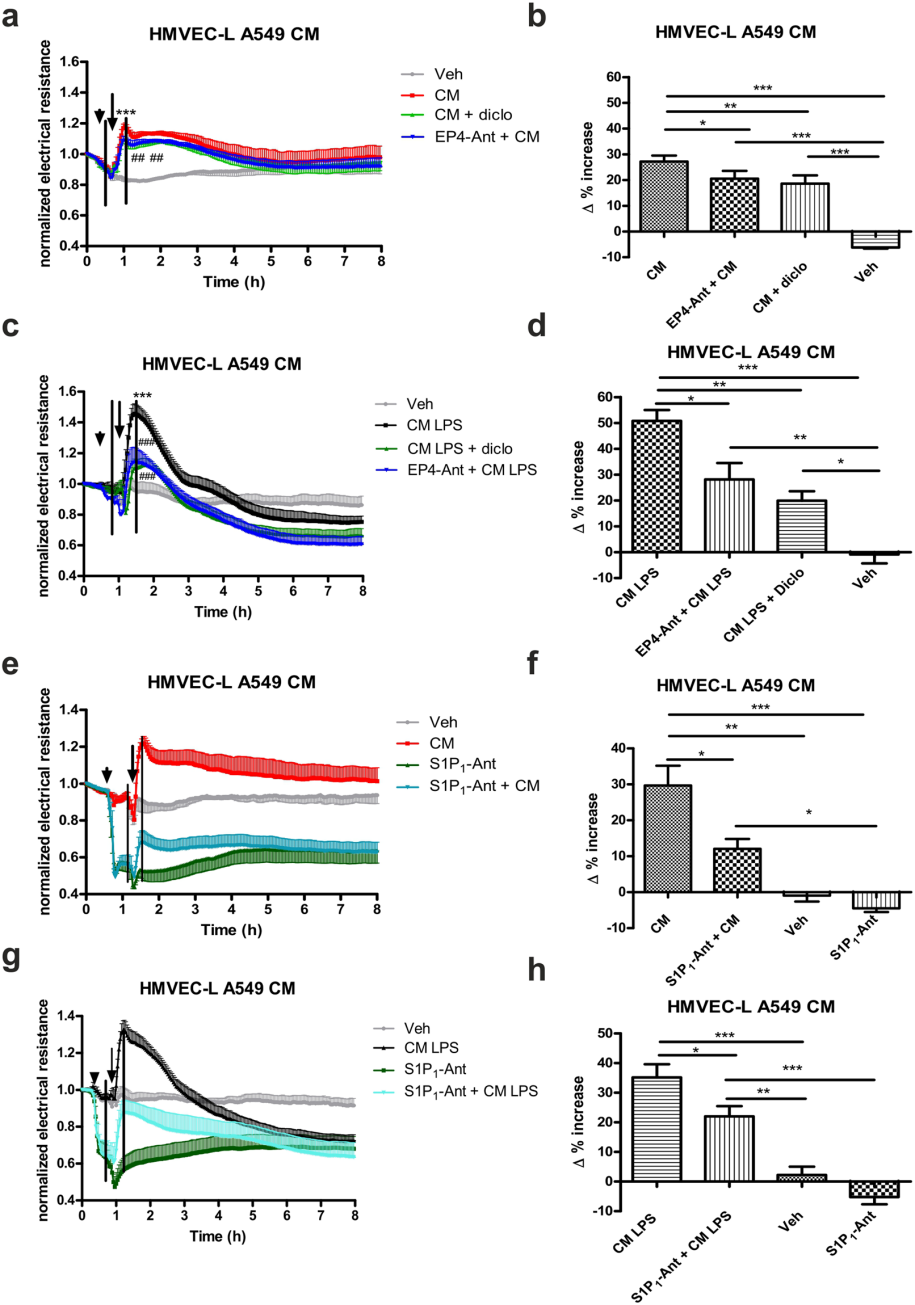
Conditioned medium (CM) from A549 cells increases endothelial barrier function via PGE_2_ and S1P_1_ receptor activation. HMVEC-L were grown on gold microelectrodes and were pretreated (arrowhead) with vehicle (Veh), the (**a,c**) EP4 antagonist ONO AE3-208 (EP4 Ant; 300 nM) or the (**e,g**) S1P_1_ antagonist W146 (S1P_1_-Ant; 10 µM). CM from (**a,e**) vehicle, (**c,g**) LPS treated (10 µg/ml) and/or diclofenac-pretreated (diclo 10 µM) A549 cells or corresponding medium (Veh) was added (indicated by arrow). To detect differences in barrier function (measured as change in resistance), Δ% values were calculated. The black lines mark the time points which were used to calculate Δ% increase shown in (**b,d,f,h**). (**a,c,e,g)** Data show mean normalized resistance + SEMs; (**b,d,f,h**) Δ% increase was calculated by subtracting normalized resistance of the first time point marked in (**a,c,e**) or (**g**) from the second time point, and multiplying the value with 100. Statistical analysis was performed using two-way ANOVA and Tukey’s multiple comparison test for (**a,c,e**) and (**g**) and one-way ANOVA followed by Bonferroni’s post hoc test for (**b,d,f)** and (**h**); n = 5–6, *p < 0.05; **p < 0.01; ***p < 0.001; * vs vehicle; # vs CM alone.

**Figure 6:**
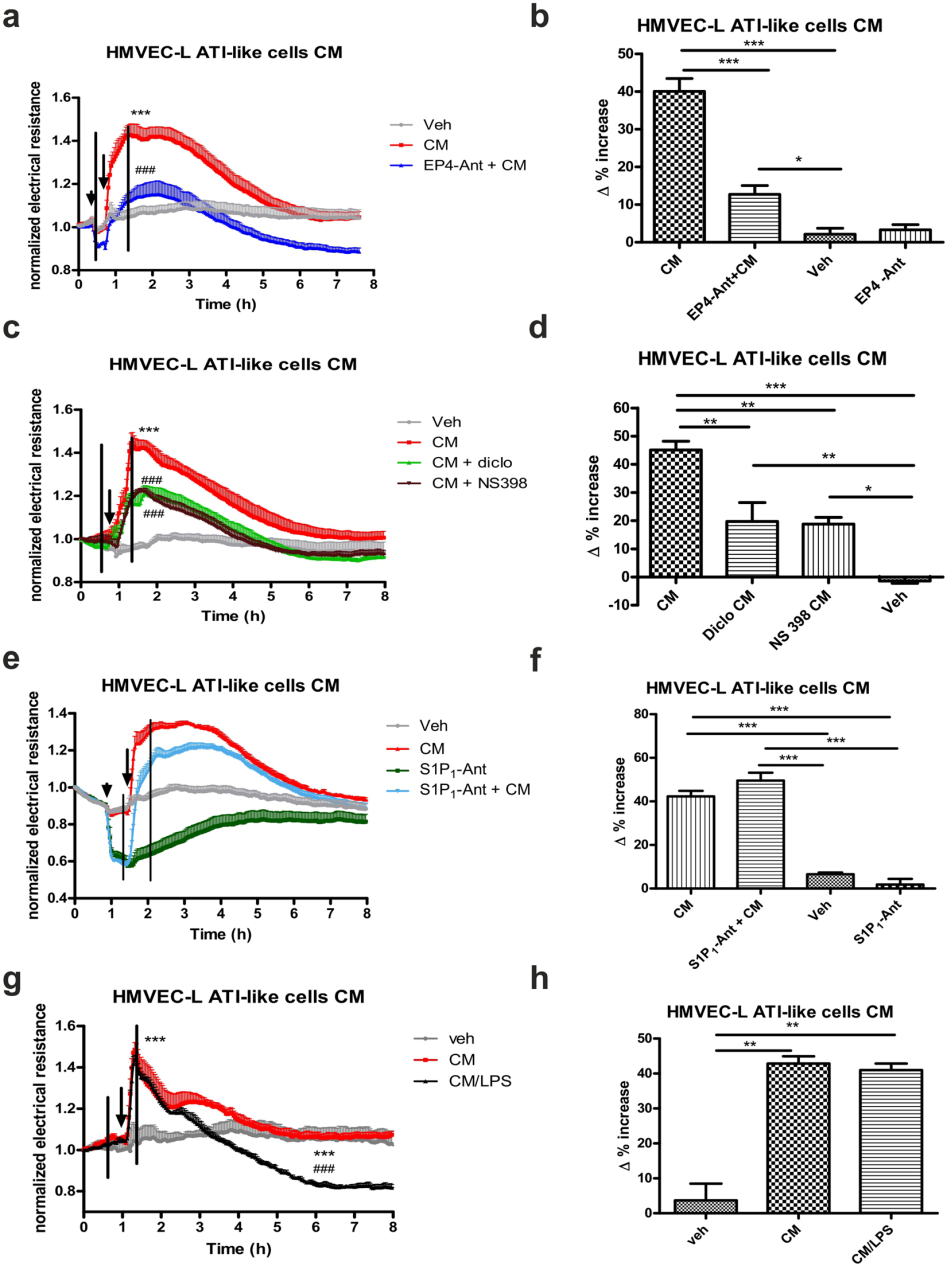
Conditioned medium (CM) from isolated mouse ATI-like cells increases endothelial barrier function via PGE_2_ and EP4 receptors but not S1P_1_ receptors. HMVEC-L were grown on gold microelectrodes and were pretreated (arrowhead) with vehicle, the (**a**) EP4 antagonist ONO AE3-208 (EP4 Ant; 300 nM) or the (**e**) S1P_1_ antagonist W146 (S1P_1_-Ant; 10 µM). CM from ATI-like cells treated with (**a**–**f**) vehicle, (**c**,**d**) diclofenac (diclo 10 µM), NS398 (1 µM) and (**g**,**h**) LPS (10 µg/ml) or corresponding medium (Veh) was added (indicated by arrows). To detect differences in barrier function (measured as change in resistance), Δ% values were calculated. The black lines mark the time points which were used to calculate Δ% increase shown in (**b,d,f,h**). (**a,c,e,g**): data show mean normalized resistance + SEMs; (**b,d,f,h**): Δ% increase was calculated by subtracting normalized resistance of the first time point marked in (**a,c,e**) or (**g**) from the second time point, and multiplying the value with 100. Statistical analysis was performed using two-way ANOVA and Tukey’s multiple comparison test for (**a,c,e**) and (**g**) and one-way ANOVA followed by Bonferroni’s post hoc test for (**b,d,f**) and (**h**); n = 5, *p < 0.05; **p < 0.01; ***p < 0.001; * vs vehicle; # vs CM alone.

Conditioned medium from A549 cells incubated for 24 hours increased the barrier function of HMVEC-L as recorded as electrical endothelial resistance. The increase in barrier function peaked at 40–45 min and remained elevated for 3 to 4 hours (Fig. 5a). The selective EP4 receptor antagonist ONO AE3–208 (300 nM) (which showed no effect on its own, data not shown) caused only a small, but significant reduction of barrier enhancement, as did the pretreatment of the epithelial cells with 10 µM diclofenac (Fig. 5a,b). In contrast, conditioned medium from LPS-stimulated A549 cells (10 µg/ml for 24 hours), which resulted in higher concentrations of PGE_2_, (cf. Fig. 1b) consistently caused a more pronounced initial barrier improvement (Fig. 5c,d). Pretreatment with diclofenac blocked PGE_2_ release from epithelial cells, and the corresponding cell supernatant caused 50% less endothelial barrier enhancement. A similar degree of reduction was achieved by pretreating endothelial cells with the EP4 antagonist ONO AE3–208 (300 nM). In contrast to the unstimulated conditioned medium, the increase in barrier function lasted only for 2 to 3 hours, after which electrical resistance dropped below baseline levels (Fig. 5c). This was most likely due to the presence of LPS in the conditioned medium, which we confirmed by adding LPS directly to the endothelial cells and observing similar decline in barrier function (cf. Fig. 6g,h).

Since the increase in barrier function attributable to PGE_2_ seemed to explain only a small part of the total effect of unstimulated conditioned medium, we checked for another lipid mediator to account for the remaining activity. Since it is known that A549 cells produce S1P^23^, which reportedly increases endothelial barrier function^19, 24^, we used the selective S1P_1_ antagonist, W146. Interestingly, W146 (10 µM) decreased the basal resistance by its own and effectively blocked the barrier enhancing response to the conditioned medium from unstimulated A549 cells (Fig. 5e,f). The barrier enhancing effect of medium from LPS- stimulated cells was also inhibited by the S1P_1_ antagonist (Fig. 5g,h). These results suggest, that while under unstimulated conditions the barrier enhancing effect seems to be mediated mainly by S1P, the increase in barrier function of conditioned medium from LPS treated cells is mediated by S1P and PGE_2_.

### Mouse primary ATI-like cell conditioned medium induces endothelial barrier enhancement via EP4 receptor activation

Next we set out to investigate the endothelial barrier promoting ability of conditioned medium from primary alveolar epithelial cells. Conditioned medium from non-stimulated primary mouse ATI-like cells caused an increase in barrier function of HMVEC-L (Fig. 6). The effect was more pronounced when compared to the effect of conditioned media from A549 cells (cf. Fig. 5). The EP4 antagonist ONO AE3-208 (300 nM) counteracted the increase in barrier function significantly at each time point measured throughout the whole experiment (Fig. 6a,b). In samples from cells that had been pretreated with diclofenac (10 µM) or NS398 (1 µM), which prevent PGE_2_ synthesis (cf. Fig. 1c,d), the barrier strengthening effect was significantly reduced (Fig. 6c,d). In contrast to conditioned medium from A549 cells, the S1P_1_ receptor antagonist W146 (10 µM) had no effect on the increase in barrier function by conditioned media from ATI-like cells (Fig. 6e,f). Conditioned media from ATI-like cells that had been stimulated with LPS (10 µg/ml) showed similar results as unstimulated conditioned media (Fig. 6g,h). This was most likely due to the fact that the maximum effect was already induced by the PGE_2_ concentrations present in conditioned medium at baseline^19^. Since we also found increased levels of the PGI_2_ metabolite 6-keto-PGF_1α,_ we were interested whether PGI_2_ adds to the barrier enhancing effect of conditioned medium of ATI-like cells. However, the stable PGI_2_ analogue iloprost (500 nM)^25^ caused only a small increase in resistance which was readily inhibited by the IP receptor antagonist Cay10441 (1 µM). The IP receptor antagonist Cay10441 (1 µM) did not significantly affect the barrier enhancement induced by conditioned medium from ATI-like cells (Supplementary Fig. S3).

### Mouse primary ATI-like cell conditioned medium protects against thrombin-induced stress fiber formation and disruption of cell junctions via EP4 receptor activation in HMVEC-L

**Figure 7:**
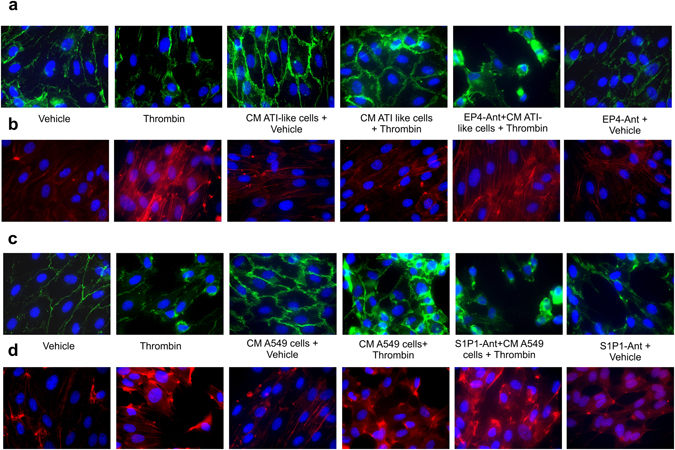
Conditioned medium protects endothelial cells from thrombin-induced disruption of adherens junctions and stress fiber formation. HMVEC-L were grown in chamber slides and pretreated with either vehicle, the EP4 antagonist ONO AE3 208 (EP4-Ant; 300 nM) or the S1P_1_ antagonist W146 (S1P1-ANT; 10 µM) as indicated. Conditioned medium (CM) from (**a**,**b**) ATI-like cells or (**c**,**d**) A549 cells was added followed by 0.5 U/ml of thrombin or vehicle for 15 min. Afterwards, cells were stained with (**a**,**c**) an antibody against VE-cadherin (green) to visualize adherens junctions and (**b**,**d**) phalloidin (red) for stress fiber formation. Nuclei were counterstained using DAPI. Images are representative for 5 different experiments.

In order to corroborate our findings concerning the barrier enhancing effects of conditioned medium, we assessed cell junctions and stress fiber formation, two hallmarks of endothelial barrier function. Addition of conditioned medium of mouse primary ATI-like cells alone resulted in enhanced VE-Cadherin staining in the junctional zone. Thrombin (0.5 U/ml) led to stress fiber formation and intercellular gaps, and this in turn was prevented by conditioned medium pretreatment. These beneficial effects were abrogated by EP4 receptor blocking with the specific antagonist ONO AE3-208 (300 nM; Fig. 7a,b). Similar effects were found with A549 cell conditioned medium, although the protection was less conclusive, being consistent with the smaller increase of electrical resistance (Fig. 7c,d c.f. Fig. 5a). The S1P_1_ receptor antagonist W146 (10 µM) on its own induced barrier disruption to some degree; however, it also abolished the protective effects of conditioned medium from A549 cells.

### LPS does not regulate EP4 receptor-expression and EP4 receptor activation restores LPS- induced disruption of barrier function on HMVEC-L

**Figure 8:**
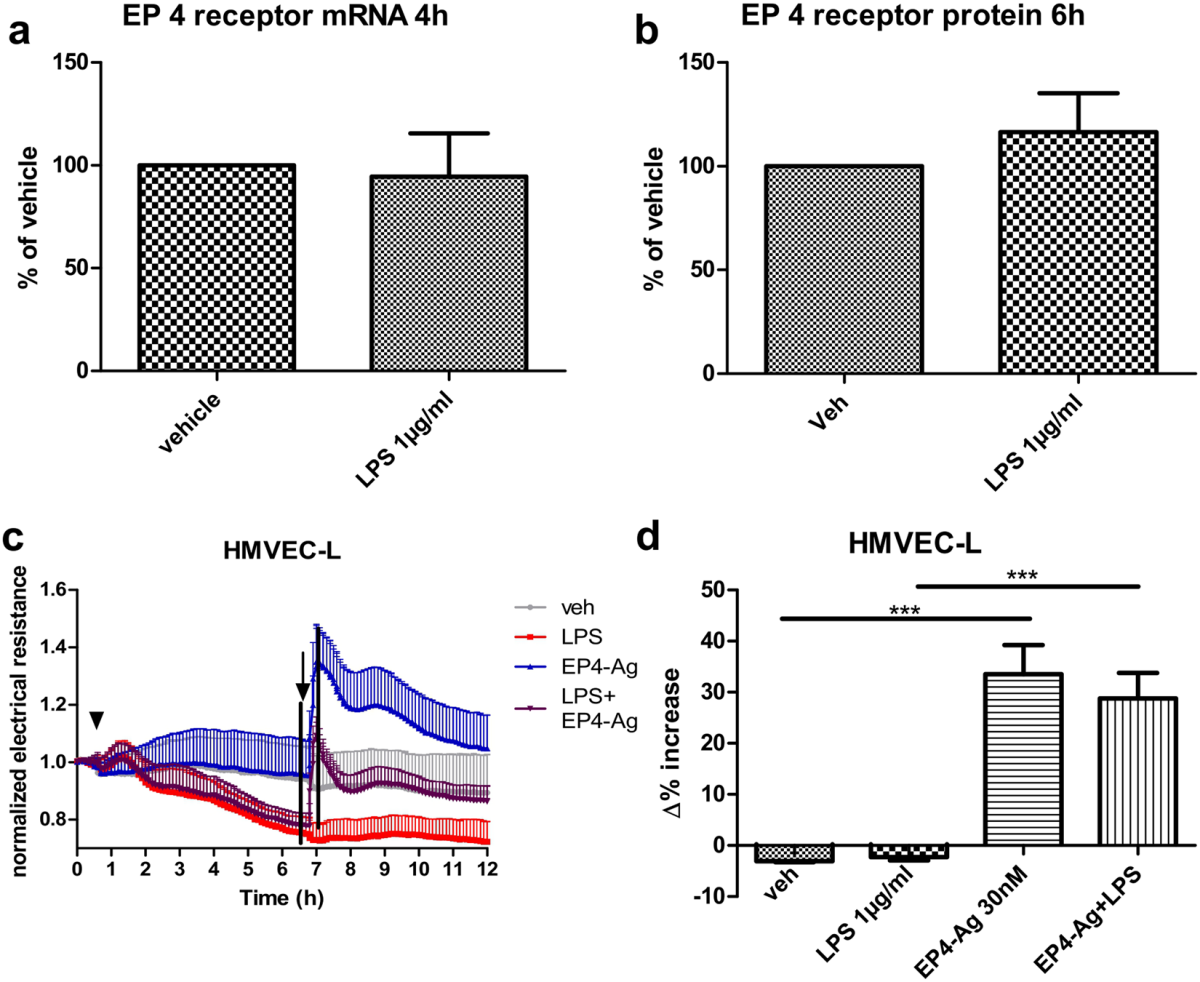
LPS treatment does not affect EP4 receptor expression and EP4 receptor activation restores LPS- induced disruption of HMVEC-L barrier function. HMVEC-L were treated with either LPS (1 µg/ml) or vehicle for (**a**) 4 and (**b**) 6 hours. (**c**,**d**) For assessment of barrier function HMVEC-L were starved for 1 hour and treated with LPS (1 µg/ml) or vehicle (arrowhead) for 6 hours. At this time point, an EP4 receptor-agonist ONO AE1 329 (30 nM) was added (arrow). To detect differences in barrier function (measured as change in resistance), Δ% values were calculated. The black lines mark the time points which were used to calculate Δ% increase shown in (**d**). (**a**) Data show the percentage of EP4 receptor mRNA as percent of vehicle control; (**b**): Data show the percentage of EP4 receptor protein as percent of vehicle control; (**c**): Data show mean normalized resistance + SEMs; (**d**): Δ% increase was calculated by subtracting normalized resistance of the first time point marked in c from the second time point, and multiplying the value with 100. Statistical analysis was performed using one sample t-test against 100 for (**a–b**) and one-way ANOVA followed by Bonferroni’s post hoc test for (**d**); n = 5–10; ***p < 0.001.

Literature indicates that the EP4 receptor is downregulated upon treatment with LPS^26^, so we analyzed HMVEC-L for their EP receptor expression patterns. As reported, we found high levels of EP4 receptor mRNA and very low levels for the EP1 receptor mRNA (data not shown), while EP2 and EP3 receptor mRNA was not detected^27^. The mRNA levels did not change after 4  hours of LPS treatment and neither did EP4 protein levels after 6 hours, two established time points to measure regulation of mRNA and proteins of EP receptors^26, 28^ (Fig. 8a,b). Furthermore, we tested the barrier function by treating HMVEC-L with LPS for 6 hours and afterwards added an EP4 receptor-agonist, ONO AE1 329 (30 nM). We found that the response was similar despite LPS treatment and that the EP4 agonist was able to restore barrier function to similar levels as seen in vehicle-treated cells (Fig. 8c,d).

## Discussion

In this study, we show that lung epithelial cells profoundly regulate the barrier function of the lung microvasculature by releasing barrier-enhancing factors, two of which we identified as PGE_2_ and S1P, acting on EP4 and S1P_1_ receptors, respectively. Upon inflammatory stimulation of epithelial cells by LPS, the barrier-enhancing activity even increases, suggesting that it represents a crucial protective mechanism of the lung against tissue damage.

Conditioned media from A549 cells, an adenocarcinoma-derived cell line, were shown to increase endothelial barrier function under basal and stimulated conditions^15^. Despite their origin, these cells are widely used as a model for distal lung epithelial cells, being described as matching an ATII-cell phenotype^29, 30^. We found that, under basal conditions, A549 cells release low levels of PGE_2_; however, treatment with LPS resulted in increased PGE_2_ release, which is in line with previous findings^31^. We showed that A549 cells express COX-2, which is upregulated upon LPS stimulation^32^, but in accordance with previous reports they do not express COX-1^33^. Therefore, PGE_2_ contributed to the barrier enhancement caused by A549 cell conditioned medium under basal conditions in a very limited fashion. This led us to further investigate other possible mechanisms involved in the described barrier enhancing effect of conditioned medium from A549 cells. Among the more prominent modulators in endothelial barrier function is the S1P_1_ receptor^24^, one of five G protein-coupled receptors for S1P. S1P has been detected in A549 cell supernatants^23^ and in our hands, the S1P_1_ antagonist markedly decreased the baseline resistance of endothelial cells. This is consistent with *in vivo* data demonstrating an enhancement of capillary leakage by S1P_1_ antagonism^34^. Therefore, it seems that S1P that is produced by endothelial cells and red blood cells, is present at high levels in the blood and is an important mediator of barrier function^35, 36^; however, we were not able to detect S1P in supernatants of A549 cells. This may be due to the different methods employed to detect S1P; while we used mass spectrometry, Schnitzer *et al*.^23^ used an ELISA. The S1P_1_ antagonist largely abolished the barrier effect of A549-conditioned medium under basal conditions. However, conditioned medium from LPS-stimulated A549 cells regulated barrier function in a different manner. First, it induced a more pronounced increase in barrier function, and second, this seemed to be due to PGE_2_, via EP4 receptor stimulation, since both diclofenac and an EP4 antagonist counteracted this effect. Although both mediators are involved in the regulation of barrier function, it is tempting to speculate that S1P is more important in maintaining the blood-air barrier under physiological conditions, while under inflammatory conditions also PGE_2_ acts as a barrier-promoting agent^20^. However, there are important limitations to this consideration. First of all, A549 is a cancer cell line and could thus show important differences as compared to primary cells, which did not rely upon S1P_1_ receptor stimulation at all. Indeed, PGE_2_, as well as S1P are thought to promote proliferation and inhibit apoptosis in A549 cells^16, 23, 37^. Furthermore, PGE_2_ levels in the lung tend to be higher than in other organs and isolated alveolar epithelial cells have been shown to release high amounts of PGE_2_^21^, which is in contrast to the low basal levels found in A549 cells. In addition, we showed recently, that PGE_2_ can be detected in the broncho-alveolar lavage from BALB/c mice^38^.

In fact, we found that in contrast to A549 cells, conditioned medium of alveolar epithelial cells isolated from BALB/c mice contained substantial amounts of PGE_2_ already under unstimulated conditions. Furthermore, we found that primary alveolar epithelial cells produce a wide variety of prostaglandins, with PGE_2_ and 6-keto-PGF_1α_, a PGI_2_-metabolite, being the most abundantly secreted ones. The relative levels of prostaglandins are in line with earlier reports showing significant production of prostanoids by rabbit alveolar epithelial cells^39^. In A549 cells, only PGE_2_ was detected. In good agreement with these findings we observed that when HMVEC-L were exposed to conditioned medium of primary alveolar epithelial cells, the increase in endothelial barrier function was more pronounced. This effect was almost completely blocked by diclofenac and NS398 as well as by a specific EP4 receptor antagonist, an observation, which highlights the importance of PGE_2_ acting on the EP4 receptor in this physiologically more relevant setting. In addition, PGI_2_ is also known to increase endothelial barrier function^25^. However, in our experimental setting iloprost showed only a small effect on endothelial barrier; therefore, we hypothesize that PGI_2_ might be more relevant in the pulmonary arteries, where we and others could show beneficial and barrier enhancing effects^25, 40^. Accordingly the barrier enhancement of conditioned medium was not affected by an IP receptor antagonist, which is probably due to the fact that PGI_2_ is rapidly hydrolized to 6-keto-PGF_1α_, which we detected in the media.

Interestingly, S1P did not seem to be involved in this setting, as the S1P_1_ receptor antagonist had no effect. This contrasting result suggested a low secretion of S1P. In contrast, PGE_2_ concentrations in conditioned media from unstimulated ATI-like cells were approximately 150-fold higher than from A549 cells. NS398 caused the same inhibition as diclofenac, suggesting involvement of a COX-2 dependent pathway. This had previously been shown for alveolar epithelial cells from C57BL/6 mice one or two days after isolation^21, 41^. To circumvent the possible induction of COX-2 caused by tissue digestion, we waited until day 6 before collecting the conditioned medium. In addition, we used dexamethasone to inhibit COX-2 expression as it destabilizes the mRNA of COX-2 and prevents its upregulation^42^. Both approaches did neither significantly influence COX-2 expression nor the levels of PGE_2_ in cell culture supernatants, supporting the view that COX-2 is constitutively expressed. As it is known that isolated mouse alveolar epithelial cells undergo transdifferentiation towards an ATI-like phenotype during culture^7^, we also investigated whether COX-2 is present in alveolar epithelial cells in the lung. It has been shown that COX-2 is expressed at high levels in lung cancer-sensitive mouse strains and to a lesser extent in resistant strains^43^, but this study did not address BALB/c mice. Furthermore, a recent study showed that COX-2 expression in lungs of BALB/c mice is restricted to bronchi and bronchiolar epithelial cells as well as to alveolar macrophages, but did not address alveolar epithelial cells^44^. In the current study, using confocal immunofluorescence microscopy, we were able to confirm COX-2 expression in alveolar epithelial cells of specific-pathogen-free BALB/c mice lungs.

The fact that the mediators that induced endothelial barrier enhancement differed in the two cell types used led us to wonder whether this S1P-like activity is limited to malignant lung epithelial cells and by secretion, to their microenvironment^23^. Although we were not able to detect S1P in A549 cell supernatants by means of mass spectrometry, others report significant production of S1P by A549 cells, high enough to explain the effects on the barrier function^23, 24^. As lysophosphatidic acid (LPA) has been described as an endogenous ligand of S1P_1_ receptor we also tested its effects on barrier function. In accordance with literature^45^, no effect of LPA on barrier function in microvascular endothelial cells was observed (see Supplementary Fig. S4). While the anti-apoptotic properties of S1P in A549 cells are known^23^, we could further show that conditioned medium from A549 cells induces S1P_1_ receptor activation on human lung microvascular endothelial cells and thereby affects barrier function. Interestingly, conditioned medium from primary ATI-like cells did not rely on S1P_1_ receptors to promote endothelial barrier function. Thus, our results urge caution when using cancer cells to model physiological processes.

There are, however, some limitations to our study. First, further investigations are needed in human primary alveolar epithelial cells, to exclude the possibility that the profile of secreted lipid mediators could differ. However, as rabbit and mouse alveolar epithelial cells show remarkable similarities in their prostanoid secretion profile, it is reasonable to expect the same ratios in human cells^39^. Although PGE_2_ is also a mediator of cancer progression and angiogenesis^16, 46, 47^, several lines of evidence show its beneficial effects in the lungs and its importance in several physiologic processes, such as maintenance of barrier integrity^18, 20^.

PGE_2_ has been shown to counteract apoptosis on endothelial cells under serum starvation^48^, a condition which we used for ECIS experiments, but the barrier enhancing effects started almost immediately after addition of conditioned medium; therefore, it seems unlikely that this mechanism contributes to the observed effects. Moreover, *in vivo* studies, e.g. by employing conditional endothelial cell-specific EP4 receptor KO mice, would further enhance our understanding of alveolar epithelial-microvascular endothelial cross talk.

Taken together, our findings provide us novel insights about the nature of epithelial-endothelial crosstalk in the lungs, as well as about the factors mediating this interaction. PGE_2_ by activating EP4 receptors is protective in the lung, which acts via promotion of endothelial barrier function, and we show that the levels of PGE_2_ secreted by primary alveolar epithelial cells in culture are actually high enough to exert this effect. Interestingly, although PGE_2_ is upregulated following LPS stimulation, LPS is still known to lead to lung injury and edema formation^12^. One possible explanation is that primary alveolar epithelial cells are limited in upregulating PGE_2_ production (c.f. Fig. 1). Furthermore, we did not observe differences in the increase in barrier function whether conditioned medium from vehicle or LPS-stimulated cells (containing more PGE_2_) was applied (c.f. Fig. 6). We therefore hypothesize that these mechanisms by themselves are not sufficient to counteract the LPS-induced injury completely. Nonetheless, prostanoids produced from alveolar epithelial cells are protective, as a recent paper addressing acute lung injury in ATII-cell-specific COX-2 knockout mice reported increased levels of neutrophils and total cell counts in the bronchoalveolar lavage-fluid^49^ and aggravated overall inflammation. Together with the fact that PGE_2_ via EP4 receptor stimulation inhibits inflammation and neutrophil influx^20, 50, 51^, we provide further evidence that PGE_2_ synthesized via the COX-2 pathway in ATII cells acts as an anti-inflammatory mediator. This is strengthened by our finding that in contrast to other cells, the EP4 receptor is not downregulated in HMVEC-L upon LPS stimulation and that EP4 receptor activation successfully restores barrier function back to baseline^26^. It is conceivable that the axis we describe herein represents a primary pulmonary defence mechanism, which is activated upon contact with gram-negative bacteria. By strengthening microvascular barrier, bacteria could be hindered from entering the blood stream and the alveoli could be protected from exaggerated inflammation. Exploiting this mechanism by using a selective EP4 agonist could thus ameliorate conditions such as ARDS.

To conclude, we demonstrate here that conditioned media from the adenocarcinoma lung epithelial cells, A549, increase the endothelial barrier function via activation of the S1P_1_ receptor under basal conditions. However, PGE_2_ seems to mediate the endothelial barrier promotion of LPS-stimulated A549 cell supernatant. Notably, the PGE_2_–EP4 axis fully accounts for the crosstalk between primary ATI-like cells and microvascular lung endothelial cells concerning endothelial barrier enhancement. Therefore, EP4 receptor activation shows great potential as a target in ARDS-therapy.

## Materials and Methods

### Reagents

Chemicals were obtained from Sigma (Vienna, Austria) unless specified otherwise. The sphingosine 1 phosphate (S1P)_1_ receptor antagonist W146, the IP receptor antagonist Cay10441 and S1P were from Cayman Chemical (Ann Arbor, MI, USA), Vectashield/DAPI mounting medium was obtained from Vector Laboratories (Burlingam, CA, USA). ONO AE1 329 and ONO AE3 208 were kind gifts from ONO pharmaceuticals (Osaka, Japan). Primary antibodies were purchased from Santa Cruz (Santa Cruz, CA, USA), Alomone Labs (Jerusalem, Israel), Seven Hills Bioreagents (Cincinnati, OH, USA), Abcam (Cambridge, UK), Jackson ImmunoResearch (Baltimore Pike, PA, USA) and Sigma as shown in Table 1. Dispase was purchased from Roche (Basel, Switzerland), and EGM-2 MV medium from Lonza (Basel, Switzerland). Dulbecco’s modified Eagle medium (DMEM) and FCS were from Gibco (Carlsbad, CA, USA). A list of antibodies used and their dilutions is shown in Table 1.

**Table 1:** Antibodies (AB) used in Western blots (WB) and immunofluorescence (IF) microscopy.

	antibody	host species	company	number	IF dilution	WB dilution
primary AB	COX2	rabbit	Abcam	15191	1:500	1:1000
	COX1	goat	Santa Cruz	sc-1754	1:250	1:200
	pro-SP-C	rabbit	Seven Hills	WRAB-9337	1:1000	—
	AQP5	rabbit	Alomone Labs	AQP-005	1:500	—
	beta-actin	mouse	Sigma	A2228	—	1:7500
	VE-cadherin	mouse	Santa Cruz	sc-9989	1:200	—
	EP4 receptor	rabbit	Abcam	133170	—	1:200
	F’ab fragments	goat	Jackson Immunoresearch	111-007-003	1:50	—
secondary AB	anti rabbit Cy3	goat	Invitrogen	A-10520	1:500	—
	anti goat AF488	rabbit	Invitrogen	A-11078	1:500	—
	anti rabbit AF488	goat	Invitrogen	A-11008	1:500	—
	anti goat AF594	donkey	Invitrogen	A-11058	1:250	—
	anti rabbit HRP	goat	Cell signaling	70745	—	1:1000
	anti goat HRP	donkey	Jackson Immunoresearch	705-035-003	—	1:1000
	anti mouse HRP	goat	Jackson Immunoresearch	115-036-062	—	1:1000

### Animals

Primary cells were isolated from specific pathogen free (SPF) 6–8 weeks old male BALB/c mice (Charles River, Sulzfeld, Germany). Mice were kept at the SPF unit of the Medical University of Graz and were accustomed to the environment for at least 1 week. All animal care complied with national and international guidelines. As lungs were harvested from previously euthanized animals, our experiments did not qualify as animal experiments and thus did not need ethical approval by an institutional committee.

### Culture of A549 and human lung microvascular endothelial cells

The human cell line A549 was purchased from Cell Line Service (Eppelheim, Germany) and cultured in DMEM supplemented with 10% FBS, penicillin (100 U/ml)/streptomycin (100 µg/ml). A549 cells were seeded in 24-well plates at a density of 10^5^ cells per well and kept for 3 days in 300 µl of medium. Thereafter, cells were starved (DMEM, 2% FBS and penicillin (100 U/ml)/streptomycin (100 µg/ml)) and pretreated with either NS398 (1 µM) or diclofenac (10 µM) for 20 min or with dexamethasone (1 µM) and corresponding vehicles for 2 days before addition of LPS (10 µg/ml; *Escherichia coli* O55:B5) or vehicle. 10 µg/ml LPS have been shown to induce maximal PGE_2_ synthesis in A549 cells and the same concentration was subsequently used for primary alveolar epithelial cells^52^. After 24 hours the conditioned medium was collected, centrifuged at 440 x g for 5 min and the supernatants were kept at -70 °C until use.

Human lung microvascular endothelial cells (HMVEC-L, Lonza, Basel, Switzerland) were cultured in EGM-2 MV medium supplemented with 5% FBS until passage 8 as described previously^19^. In order to disrupt barrier function, LPS was added at a concentration of 1 µg/ml^20^.

### Isolation and culture of primary mouse alveolar epithelial cells

Mouse alveolar epithelial cells were isolated from the lungs of specific pathogen free BALB/c mice as described previously^7, 53, 54^. Mice were euthanized with an overdose of pentobarbital (150 mg/kg i.p.), exsanguinated and the lung was perfused with HEPES buffered saline solution. After removing the lung and heart *en bloc*, 1 ml of dispase (50 U/ml) followed by 0.5 ml of low-melting agarose were instilled via a tracheal cannula and the lung was placed on ice. When the agarose had hardened, the lungs were transferred into a 50 ml Falcon tube, containing dispase solution. The lungs were incubated for 40 min at room temperature and minced thoroughly. The resulting single cell suspension was subjected to negative magnetic separation with the EasySep-kit (StemCell, Vancouver, CA). Cells were then seeded at a density of 10^6^ cells/cm² in 48-well plates coated with laminin 1 (50 µg/ml). Epithelial cell medium (ECM) with Bullet Kit, containing 5% FCS, antibiotics/antimykotics, EGF, insulin transferrin sodium, L-Glutamine and hydrocortisone (Cell Biologics, Chicago, IL, USA) was first changed on day 3, and afterwards every day. Cells were allowed to grow for 6 days, until they showed an ATI-like phenotype as previously described^7^ which was confirmed by aquaporin (AQP)5-staining (see Supplementary Fig. S2). On day 6, cells were treated with either LPS 10 µg/ml or vehicle for 8 hours. Thereafter, the conditioned medium was collected, centrifuged to remove cell debris (440 × g, 5 min) and the supernatants were frozen at −70 °C for subsequent experiments. In separate groups of experiments, cells were pretreated with either NS398 (1 µM) or diclofenac (10 µM) for 20 min or for 3 days (starting on the 3^rd^ day) with dexamethasone (1 µM) and corresponding vehicles, before addition of LPS or vehicle. For experiments including dexamethasone, hydrocortisone was omitted from the ECM.

### Endothelial electrical resistance measurements

HMVEC-L (8 × 10^4^ per chamber) were seeded on 1% gelatin-coated biochips with gold electrodes 8W10E + (Applied Biophysics, Troy, NY, USA) and grown to confluence for two days. On the day of the experiment, the cells were starved in medium containing 2% FBS for 1 hour, afterwards the electrical resistance of the cell monolayers was measured at a frequency of 4000 Hz using Electrical Cell-Substrate Impendance Sensing (ECIS; Applied Biosphysics) system, as previously described^19, 20^.

### Real-time polymerase chain reaction

For relative quantification of mRNA real-time PCR was performed as described previously (CDX Connect^TM^ Real-Time PCR detection system with CFX Manager^TM^ software 3.1; Biorad, Hercules, CA, USA))^27^. Four hours after LPS or vehicle treatment, RNA isolation was performed using TRIzol (Thermo Fisher Scientific, Waltham, MA, USA) and DNA removal was performed with Ambion DNA removal kit (Thermo Fisher Scientific). 1 µg of total RNA was reverse transcribed using the iScript cDNA Synthesis Kit (Biorad) according to the manufacturer’s instruction. Real-time PCR was performed using SsoAdvanced™ Universal SYBR® Green Supermix with PrimePCR™ SYBR® Green Assay primers for PTGER4 or GAPDH, human (both Biorad) according to manufacturer’s instructions. Samples were measured in triplicates and GAPDH was used as the reference gene. Quantification of mRNA expression relative to vehicle were calculated with the 2^−ΔΔCT^ method and data are shown as percentage of vehicle.

### Western blot

Western blots were performed as described^55^. Briefly, HMVEC-L, ATI-like cells or A549 cells were mechanically detached by adding extraction buffer (50 mM TRIS, 10 mM EDTA, 1% v/v Triton X, 1 mM PMSF and proteinase inhibitor cocktail) to each well. The samples were analyzed for their protein content by using the Pierce BCA-kit (Thermo Fisher Scientific, Waltham, MA, USA), and 15 µg protein per sample were separated by SDS-PAGE on a 4–20% TRIS-glycine gradient gel (Thermo Fisher Scientific). The protein was blotted onto a polyvinylidene fluoride membrane (Bio-Rad, Vienna, Austria). Target proteins were immunochemically detected using specific antibodies for EP4 receptor, COX-2 or COX-1 and visualized with the respective horseradish peroxidase (HRP) conjugated antibodies (Table 1) and HRP- detection substrate (Bio-Rad). Chemoluminescence was recorded by ChemiDoc Touch Imaging system (Bio-Rad). After stripping the membranes by adding 62.5 mM TRIS HCl pH = 6.8, 2% SDS, 100 mM β-mercaptoethanol at 55 °C for 30 min, membranes were reprobed with beta-actin antibody and further processed as described above. Densitometric analysis of protein bands was performed using Imagelab Software (Bio-Rad).

### Immunofluorescence staining

#### Mouse lung sections

Five-µm sections of paraformaldehyde-fixed and paraffin-embedded mouse lungs were rehydrated and afterwards subjected to antigen-retrieval procedure (2 × 5 min in microwave, 750 W in 10 mM trisodium citrate, pH = 6). In order to reduce autofluorescence, sections were immersed in Sudan Black B (0,3% in 70% ethanol), quenched with glycine (1 mg/ml) and sodium borohydride (1 mg/ml)^56, 57^. After blocking with 4% BSA, 2% serum and 0.3% Triton X, slides were incubated with the specific antibodies for COX-1 or COX-2 (Table 1) overnight at room temperature. The next day, the secondary antibodies (goat anti-rabbit IgG, or donkey anti-goat IgG, (Table 1)) were applied for 2 hours. Thereafter, slides were incubated with rabbit serum, followed by addition of Fab fragments of a goat anti- rabbit antibody. Afterwards, the slides were transferred to a microwave oven and heated in trisodium citrate buffer (10 mM sodium tri-citrate, pH = 6.0) for 3 × 5 min, at 750 W and finally left to cool down^58^. Incubation with pro-surfactant protein-C (SP-C)-antibody was also performed overnight. Alexa Fluor (AF) 488 goat anti-rabbit was used as a secondary antibody and slides were then mounted with DAPI-containing mounting medium (Vectashield) and sealed with nail polish. Appropriate isotype control antibodies were used and omission of the primary antibody was performed as controls in each experiment. Sections were analyzed with a confocal laser scanning microscope (Zeiss LSM 510 META, with a Plan-Neo 63x/1.4 Oil with DIC capability lens) and processed with ZEN software (Zeiss, Jena, Germany) and ImageJ^59^. An UV 405 nm (DAPI), a tunable Argon 458/477/488/514 nm (AF488) and a Helium-Neon 543 nm (Cy3 and AF594) laser were used. Brightness and contrast of the photomicrographs were adjusted using ImageJ^60^. For quantification of colocalisation, total cell numbers (nuclei) and cells which stained positive for COX-1, COX-2 or pro-SP-C were counted manually and percentages of cells which stained positive for respective markers were calculated for at least 5 different images from each mouse (n = 4).

#### Chamber slides

Primary mouse ATI-like cells were seeded at a density of 10^6^ cells/cm² in laminin 1-coated chamber slides (Thermo Fisher Scientific). On day 6 cells were washed thoroughly, fixed and permeabilized with methanol (for COX-2) or acetone (for COX-1). After blocking, cells were incubated with antibodies against COX-1 and COX-2 for 1 hour at room temperature. Afterwards, secondary antibodies (Table 1) were added. When double staining was performed, cells were blocked with 1% rabbit serum followed by incubation with goat anti-rabbit Fab fragments. The cells were then incubated over night with an AQP5-antibody at 4 °C, followed by incubation with a secondary antibody labeled with AF488 for 1 hour. Cells were washed and cover-slipped with Vectashield/DAPI mounting medium and examined under a fluorescence microscope (Olympus IX70) equipped with an Olympus Plan APO 60/1,42 oil lens (Olympus, Hamburg, Germany). Appropriate isotype control antibodies were used and omission of the primary antibody was performed as controls in each experiment. Photographs were taken with an ORCA-ER digital camera (Hamamatsu Photonics, Hamamatsu City, Japan) and processed by CELL P software (Olympus). Brightness and contrast of the photomicrographs were adjusted using ImageJ. For quantification of colocalization, total number of cells (nuclei) and cells which stained positive for COX-1, COX-2 or AQP5 were counted manually and percentages of cells which stained positive for according markers were calculated from at least 4 different images.

#### Endothelial cells

HMVEC-L were grown to confluence in chamber slides and pretreated with antagonist/vehicle (20 min) and conditioned medium/vehicle medium (15 min). Then thrombin (0.5 U/ml) was added for 15 min, thereafter cells were fixed with formaldehyde (3.7% for 10 min), washed and permeabilized with Triton X -100 (0.1% for 15 min). They were stained for VE-Cadherin and F-actin (Texas-Red X Phalloidin, 5 U/ml, Invitrogen) to visualize cell-adhesions and stress fiber formation, as described previously^61^. Cells were washed and cover-slipped with Vectashield/DAPI mounting medium and examined under a fluorescence microscope (Olympus IX70) equipped with an Olympus Plan APO 60/1,42 oil lens (Olympus, Hamburg, Germany).

### Radioimmunoassy

Radioimmunoassay for the determination of PGE_2_ was performed as previously described^62^. IC_50_ of PGE_2_ was 106.2 ± 10.6 pg/ml (n = 10) and detection limit, defined as 10% inhibition of binding was at 11.2 ± 1.2 pg/ml (n = 10).

### High performance liquid chromatography tandem mass spectrometry

#### Sphingolipids

Quantification of sphingolipids was done in principle as described previously^63^, except that sample size was 100 µl supernatant and sphingosine-d7, sphingosine-1-phosphate-d7 and sphinganine-d7 (250 ng/ml) were used as internal standards. The injection volume was 10 μl. In brief, the analytes were separated using a Luna C18 column (150 mm × 2 mm ID, 5 μm particle size, 100 Å pore size; Phenomenex, Aschaffenburg, Germany) under gradient conditions. The MS/MS analyses were performed using a triple quadrupole mass spectrometer API4000 (Sciex, Darmstadt, Germany) equipped with a Turbo-V-source operating in positive electrospray ionization (ESI) Multiple Reaction Monitoring (MRM) mode.

#### Prostanoids

Prostanoids were quantified as described previously^64^. In brief, 200 µl supernatant were spiked with the isotopically labeled internal standards and extracted using ethyl acetate. The chromatographic separation of the analytes was carried out using a chiral column Lux Amylose-2 (150 × 2 mm I.D., 3 µm) coupled to a Synergi Hydro-RP column (150 × 2 mm I.D., 4-µm; both from Phenomenex, Aschaffenburg, Germany) under gradient conditions. Water and acetonitrile, both containing 0.1% formic acid were used as mobile phases and sample run time was 22 min. The MS/MS system consisted of a hybrid triple quadrupole-ion trap mass spectrometer QTrap 5500 (Sciex, Darmstadt, Germany) equipped with a Turbo-V-source operating in negative ESI mode. Analysis was done in MRM mode with a dwell time of 50 ms for all analytes.

For both sphingolipids and prostanoids, data was acquired using Analyst Software V 1.6 and quantification was performed with MultiQuant Software V 3.0 (both Sciex, Darmstadt, Germany), employing the internal standard method (isotope dilution mass spectrometry). The coefficient of correlation was at least 0.99. Variations in accuracy were less than 15% over the whole range of calibration, except for the lower limit of quantification, where a variation in accuracy of 20% was accepted.

### Data analysis

Statistical analysis was performed using Graph Pad Prism® 6 (GraphPad Software, Inc. CA, USA). For electrical resistance measurements, statistical differences between the groups were determined by using Two-Way ANOVA with Tukey’s multiple comparisons test. All other comparisons were performed using One-Way ANOVA followed by Bonferroni’s post-hoc test or two-tailed t-test. Significance was set at p < 0.05. Data are given as mean ± standard error of the mean (SEM) in text and mean + SEM in the figures. Where applicable, normality was confirmed by using Kolmogorov-Smirnov testing.

## Electronic supplementary material

Supplementary Material
