# Supplementary material for: The Role of PGE2 in Alveolar Epithelial and Lung Microvascular Endothelial Crosstalk
Source: Sci Rep. 2017 Aug 11;7:7923. doi: 10.1038/s41598-017-08228-y (PMC5554158; doi:10.1038/s41598-017-08228-y)
Supplement: Supplementary file 1 — Supplementary Material [file 41598_2017_8228_MOESM1_ESM.pdf]

# Supplementary Material

The Role of $PGE_2$ in Alveolar Epithelial and Lung Microvascular Endothelial Crosstalk

Thomas Bärnthaler[1], Jovana Maric[1], Wolfgang Platzer[1], Viktoria Konya[1], Anna Theiler[1], Carina Hasenöhrl[1], Benjamin Gottschalk[2], Sandra Trautmann[4] , Yannick Schreiber[5], Wolfgang F. Graier[2], Rudolf Schicho[1], Gunther Marsche[1], Andrea Olschewski[3], Dominique Thomas[4], Rufina Schuligoi[1], Akos Heinemann[1]

[1]Institute of Experimental and Clinical Pharmacology, Medical University of Graz

[2]Institute of Molecular Biology and Biochemistry, Medical University of Graz

[3]Ludwig Boltzmann Institute Lung Vascular Research, Graz, Austria

[4]Pharmazentrum Frankfurt/ZAFES Institute of Clinical Pharmacology, Goethe-University Frankfurt

[5] Fraunhofer Institute for Molecular Biology and Applied Ecology IME, Project Group TMP, Frankfurt, Germany

**Content:**

- **Supplementary figures**
  - **Fig S1.** ***Primary mouse ATI-like cells express COX-1***.
  - **Fig S2.** ***Cells isolated from murine lungs and cultured for 6 days on laminin 1 express AQP5***
  - **Fig S3.** ***Conditioned medium (CM) from isolated mouse ATI-like cells increases endothelial barrier function independent of IP receptor activation***
  - **Fig S4.** ***LPA does not alter endothelial barrier function.***

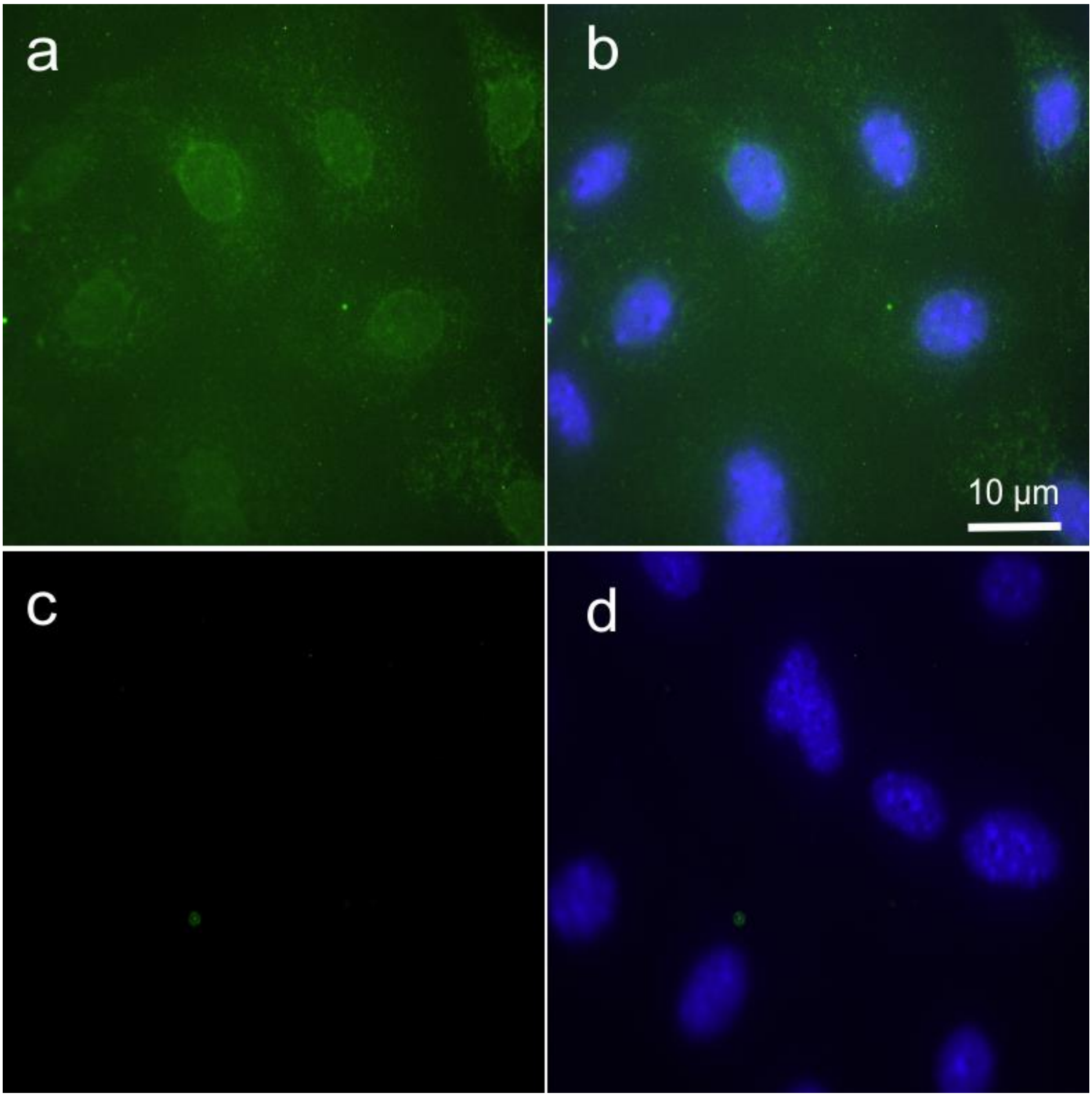


***Fig S1. Primary mouse ATI-like cells express COX-1***. *Cells grown in chamber slides for 6 days were stained with an antibody directed against (**a**) COX-1 (green) (**c**) or a matching*

*isotype control. Samples were counterstained with DAPI to reveal localization of nuclei;* ***b*** *and d shows the overlay. Images are representative for 5 independent experiments.*

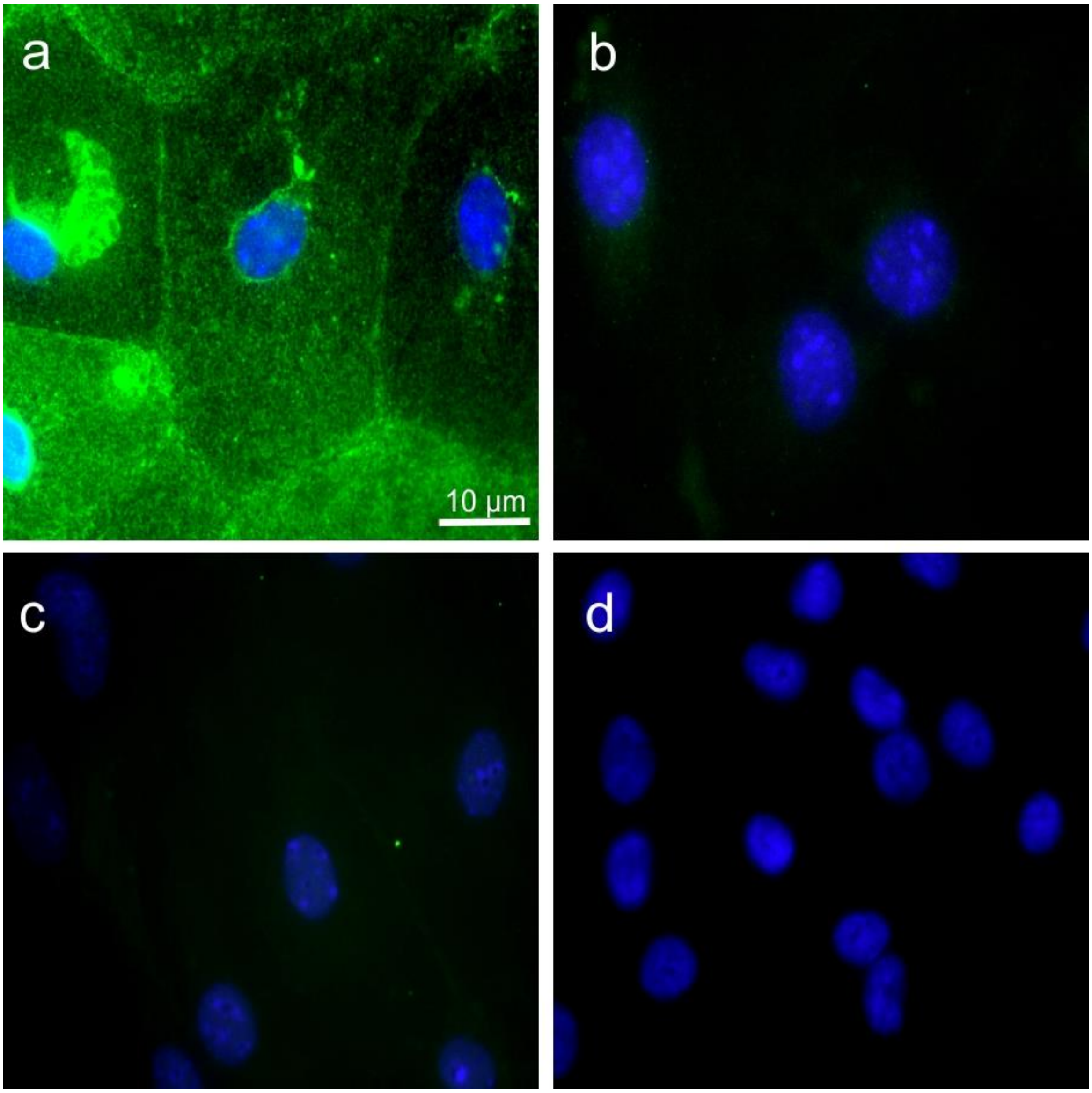


***Fig S2. Cells isolated from murine lungs and cultured for 6 days on laminin 1 express AQP5. a*** *shows AQP5-positive isolated murine ATI-like cells. Controls included (**b**) pre-incubation of antibody with blocking peptide, (**c**) incubation of cells with a matching isotype control and (**d**) a negative control using murine endothelial cells. Images are representative for 5 independent experiments.*

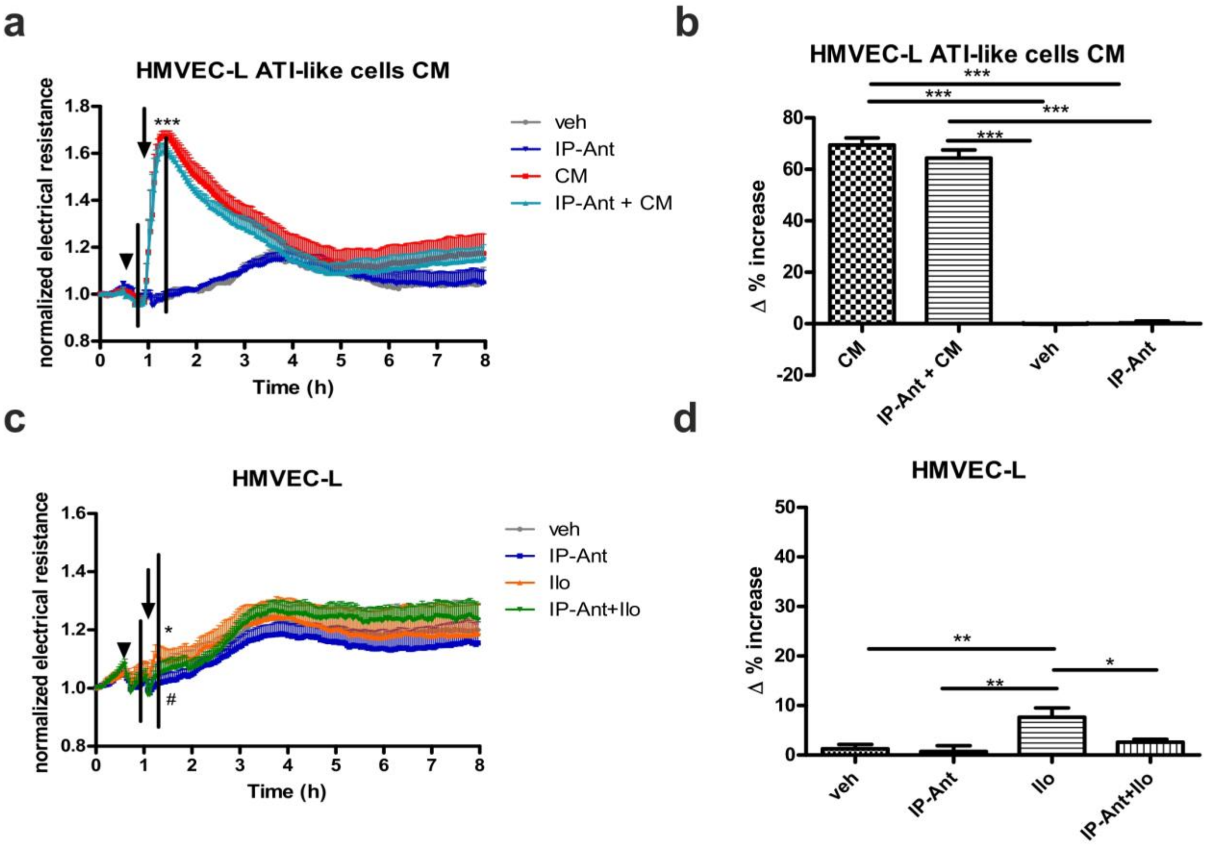


***Fig S3. Conditioned medium (CM) from isolated mouse ATI-like cells increases endothelial barrier function independent of IP receptor activation.*** *(**a, c**) HMVEC-L were grown on gold microelectrodes and were pretreated (arrowhead) with vehicle (Veh), or the IP antagonist Cay10441 (IP-Ant; 1 µM). (**a**) CM from ATI-like cells or (**c**) iloprost (Ilo; 500 nM) were added (indicated by arrow). To detect differences in barrier function (measured as change in resistance), Δ% values were calculated. The black lines mark the time points which were used to calculate Δ% increase shown in (**b, d**). **a, c**: Data show mean normalized resistance + SEMs; **b, d**: Δ% increase was calculated by subtracting normalized resistance of the first time point marked in **a** and **c** from the second time point, and multiplying the value with 100. Statistical analysis was performed using two-way ANOVA and Tukey's multiple comparison test for **a** and one-way ANOVA followed by Bonferroni's post hoc test for **b**; n=4, * $p<0.05$; ** $p<0.01$; *** $p<0.001$; * vs vehicle; # antagonist + iloprost vs iloprost alone.*

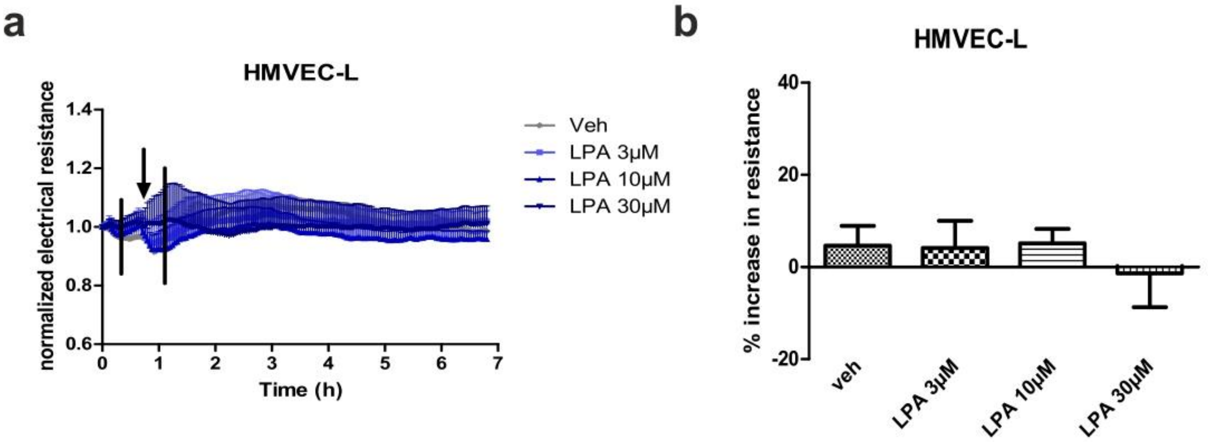


***Fig S4. LPA does not alter endothelial barrier function.*** *HMVEC-L were grown on gold microelectrodes and were treated with vehicle or increasing concentrations of lysophosphatidic acid (LPA) (arrow). To detect differences in barrier function (measured as change in resistance), Δ% values were calculated. The black lines mark the time points which were used to calculate Δ% increase shown in (**b**).* ***a****: Data show mean normalized resistance + SEMs;* ***b****: Δ% increase was calculated by subtracting normalized resistance of the first time point marked in* ***a*** *from the second time point, and multiplying the value with 100. Statistical analysis was performed using two-way ANOVA and Tukey's multiple comparison test for* ***a*** *and one-way ANOVA followed by Bonferroni's post hoc test for* ***b****; n=3. Significance was not detected.*
